# Monthly Record of Deaths in the City of Buffalo

**Published:** 1858-02

**Authors:** C. L. Dayton

**Affiliations:** Health Physician


ART. IX.— *Report of Mortality in Buffalo for the month of Dec.*, 1857. By C. L. DAYTON, M. D., Health Physician.

| DISEASES. | No. | Males. | Females. |
|---|---|---|---|
| Asthma et Diarrhœa, | 1 | 1 | |
| Accidents, | 6 | 5 | 1 |
| Atelectasis Pulmonum, | 1 | 1 | |
| Bronchitis, | 4 | 3 | 1 |
| Croup, | 4 | 3 | 1 |
| Carcinoma, | 3 | 2 | 1 |
| Cholera Infantum, | 2 | | 2 |
| Diarrhœa, | 2 | 1 | 1 |
| Debilitas Senilis, | 1 | | 1 |
| Epilepsy, | 1 | | 1 |
| Eclampsia, | 10 | 6 | 4 |
| Enteritis, | 1 | | 1 |
| Fracture, | 1 | 1 | |
| Febris Typhoid, | 6 | 5 | 1 |
| " Scarlatina, | 2 | 1 | 1 |
| " Puerperarum, | 1 | | 1 |
| Hydrops, | 3 | 1 | 2 |
| Hernia, | 3 | 3 | |
| Hydrocephalus, | 1 | 1 | |
| Hyperæmia Pulmonalis, | 2 | 2 | |
| Hæmorrhage, | 1 | | 1 |
| Intemperance et Delirium, | 2 | 2 | |
| Ignotus, | 23 | 14 | 9 |
| Marasmus, | 5 | 5 | |
| Morbus Cordis, | 1 | 1 | |
| Pertussis, | 3 | 1 | 2 |
| Pyelitis, | 1 | 1 | |
| Paralysis, | 1 | 1 | |
| Pneumonitis, | 11 | 7 | 4 |
| Parturitio, | 1 | | 1 |
| Peritonitis, | 1 | | 1 |
| Rheumatism, | 1 | 1 | |
| Rubeola, | 4 | 3 | 1 |
| Syphilis, | 1 | | 1 |
| Still-born, | 7 | 6 | 1 |
| Scrophula, | 1 | 1 | |

### REGISTER OF MORTALITY—Continued.

| DISEASES. | No. | Males. | Females. |
|---|---|---|---|
| Suicide, | 1 | 1 | |
| Tumor, | 1 | 1 | |
| Tuberculosis Pulmonalis, | 16 | 10 | 6 |
| Total, | 137 | | |

### SEXES.

| | |
|---|---|
| Males, | 91 |
| Females, | 46 |
| Sex not given, | 0 |
| Total, | 137 |

### AGES.

| | | | |
|---|---|---|---|
| Still-born, | 7 | Between 20 years and 30 years, | 15 |
| 1 day, | 4 | " 30 " " 40 " | 14 |
| 1 day and 30 days, | 10 | " 40 " " 50 " | 9 |
| Between 1 month and 6 months, | 21 | " 50 " " 60 " | 6 |
| " 6 months and 12 " | 10 | " 60 " " 70 " | 3 |
| " 1 year " 3 years, | 25 | " 70 " " 80 " | 3 |
| " 3 " " 5 " | 2 | " 80 " " 90 " | 0 |
| " 5 " " 10 " | 2 | " 90 " " 100 " | 0 |
| " 10 " " 20 " | 6 | " 100 " | 0 |
| Total number under 10 years, | 81 | Total number over 10 years, | 56 |

Ages not given, 0 Total, 137

### NATIVITIES.

| | | | |
|---|---|---|---|
| American, (white,) | 57 | Prussian, | 0 |
| " (colored,) | 1 | Swiss, | 0 |
| German, | 30 | French, | 3 |
| Irish, | 19 | Scotch, | 0 |
| English, | 5 | Poland, | 0 |
| Canadian, | 1 | Country not given, | 21 |

Total, 137

